# Silk Spinning in Silkworms and Spiders

**DOI:** 10.3390/ijms17081290

**Published:** 2016-08-09

**Authors:** Marlene Andersson, Jan Johansson, Anna Rising

**Affiliations:** 1 Department of Anatomy, Physiology and Biochemistry, Swedish University of Agricultural Sciences, Uppsala 75651, Sweden; marlene.andersson@slu.se (M.A.); janne.johansson@ki.se (J.J.); 2 Department of Neurobiology, Care Sciences and Society (NVS), Karolinska Institutet, Stockholm 14157, Sweden

**Keywords:** spidroin, fibroin, *Bombyx mori*, major ampullate gland, carbonic anhydrase, pH gradient, protein conformation

## Abstract

Spiders and silkworms spin silks that outcompete the toughness of all natural and manmade fibers. Herein, we compare and contrast the spinning of silk in silkworms and spiders, with the aim of identifying features that are important for fiber formation. Although spiders and silkworms are very distantly related, some features of spinning silk seem to be universal. Both spiders and silkworms produce large silk proteins that are highly repetitive and extremely soluble at high pH, likely due to the globular terminal domains that flank an intermediate repetitive region. The silk proteins are produced and stored at a very high concentration in glands, and then transported along a narrowing tube in which they change conformation in response primarily to a pH gradient generated by carbonic anhydrase and proton pumps, as well as to ions and shear forces. The silk proteins thereby convert from random coil and alpha helical soluble conformations to beta sheet fibers. We suggest that factors that need to be optimized for successful production of artificial silk proteins capable of forming tough fibers include protein solubility, pH sensitivity, and preservation of natively folded proteins throughout the purification and initial spinning processes.

## 1. Introduction

Spiders and silkworms produce silks with impressive properties. Not only do silk fibers represent the strongest fibers in nature [1], they are also well tolerated when implanted in pigs [2], sheep [3], and rats [4], and therefore represent interesting materials for a wide variety of applications. Spider and silkworm silks are produced in specific glands from unique proteins that are spun under ambient conditions. The domesticated silkworm *Bombyx mori* produces large amounts of silk for cocoons used during the metamorphosis from larvae to moths, while spiders spin silk for a variety of purposes, including web building, prey swathing, and reproduction, but only in small amounts. The ability to produce silk has apparently evolved multiple times, in silkworms and spiders but also in other members of the arthropod phylogeny [5]. The last common ancestor of silkworms and spiders lived around 500 million years ago [6] and although the origin of their silk producing organs is different, there are remarkable similarities in the way spiders and silkworms spin their silk, which are discussed herein.

## 2. Similar Gland Morphologies in Silkworms and Spiders

**Figure 1:**
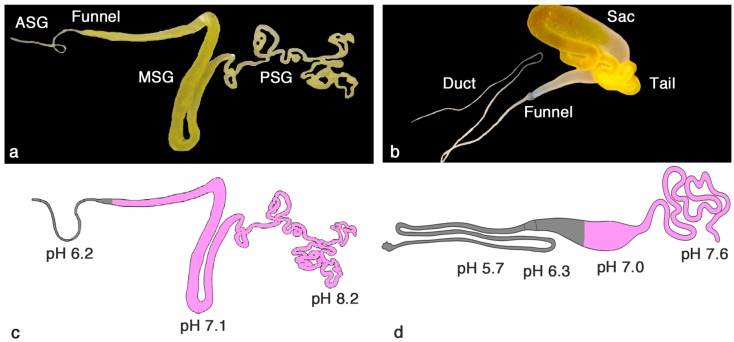
Macroscopic appearance of a *B. mori* silk gland with anterior silk gland (ASG), Funnel, middle silk gland (MSG) and posterior silk gland (PSG) identified (**a**) and a Major ampullate gland with Duct, Funnel, Sac and Tail indicated (**b**); Schematic image of a *B. mori* silk gland (**c**) and major ampullate gland (**d**) with pH values indicated in different parts. In (c) and (d) the regions containing epithelial cells with CA activity are shaded in grey, and fibroin/spidroin secreting parts are purple. Adapted from [8] (**a**,**c**) and [9,10] (**b**,**d**).

The silk glands in *B. mori* originate from salivary glands, which explains why the silk fiber exits through a pore on the lower lip of the mouth of the silkworm. The silk glands are paired, but the two filaments produced are fused into a single fiber near the pore [7]. The *B. mori* silk gland can be divided into three macroscopic parts: the posterior silk gland (PSG), the middle silk gland (MSG) and the anterior silk gland (ASG) (Figure 1a) [7,8].

In contrast to silkworms, most spiders have several different types of glands, which produce silks with varying mechanical properties [11,12]. In spiders, the silk glands are thought to have evolved from epidermal invaginations of the opisthosoma (abdomen), hence the spigots are positioned in a caudal ventral position on the opisthosoma [11]. Herein, we focus on the major ampullate gland, which produces the dragline silk. The major ampullate gland is present in pairs and is, similarly to the silkworm silk gland, divided into three parts; the tail, the sac and the duct (Figure 1b) [9,13].

The cells in the PSG of the *B. mori* silk gland, and the corresponding tail of the major ampullate gland, produce proteins—fibroins and spidroins, respectively—that are stored in the lumen of the MSG/sac [13,14] in a soluble state at a very high concentration [15]. The silk proteins are then transported through the ASG/duct where they change conformation in response to several factors (cf. below). Upon spinning, the fiber is pulled out by the motion of the head of the silkworm or by the motion of the spider’s leg or by other means (e.g., gravity if the spider is suspended, movement of the spider in the web, wind) [16].

Both the *B. mori* silk gland and the major ampullate gland epithelium consist of different types of single columnar cells in the PSG/tail and MSG/sac, respectively. The cells in the most proximal parts of the glands are responsible for secreting the major constituent of the silk, fibroins/spidroins (purple in Figure 1c,d) [8,9,17]. The cells in the MSG of *B. mori* glands secrete different types of sericin (purple, Figure 1c) [7,8,14], which will form a coat surrounding the fibroin. In the major ampullate gland, the cell types present in the proximal part of the sac produce spidroins (purple, Figure 1d) [9] while the epithelium of the most distal part of the sac and the duct produces carbonic anhydrase (CA) (grey, Figure 1d). CA interconverts H_2_O + CO_2_ and H^+^ + HCO_3_^−^ and is responsible for the generation and maintenance of the pH gradient of the gland (cf. below) [10]. In analogy, the final cell type in the MSG and the cells along the ASG found in *B. mori* silk glands likewise produce CA (grey, Figure 1c) [8].

**Figure 2:**
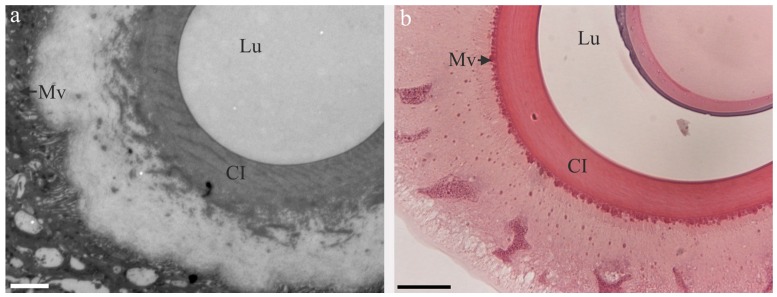
Transmission electron micrograph of a cross-section of the third limb of the duct of an *Euprosthenops australis* major ampullate gland (**a**) and a hematoxylin-eosin stained light micrograph of a cross-section of the ASG from a *B. mori* silk gland (**b**). Mv: microvilli, Lu: lumen, CI: Cuticular intima. Scale bars (**a**) 2 µm (**b**) 15 µm. In (**a**) the microvilli appear detatched from the cuticular intima, likely due to processing of the section for transmission electron microscopy.

At the funnel, a cuticular intima appears that lines the apical (interior) part of the ASG/duct epithelium all the way to the lip pore/spigot [7,8,9]. The cuticular intima in spiders has been hypothesized to protect the underlying cells of the duct from laceration caused by the appearing fiber [18]. It has also been suggested to act as a hollow dialysis membrane, aiding in dehydrating the spinning dope [19]. The ASG in *B. mori* and duct of the major ampullate gland are very similar in appearance and in a cross-section one can see epithelial cells with very abundant microvilli at the apical cell membrane, lined by the cuticular intima surrounding a lumen [8,9] (Figure 2). The diameter of the lumen in both the ASG and the duct is narrowing towards the lip pore/spigot. However, there is a big difference in size and geometry between spiders and silkworms, which is modeled to affect shear forces generated within the ASG/duct, cf. below [20]. The duct of the major ampullate gland has a slow and steady decrease in diameter going from around 100 µm to <10 µm [21], while the silkworm silk gland ASG diameter starts at 400 µm and after a sudden drop to 100 µm there is a slow decrease in diameter until it ends at around 50 µm [7].

## 3. pH Gradients, Ions and Shear Forces Govern Silk Formation

pH gradients have been inferred to be important for fiber formation in both silkworms and spiders, based on in vitro studies of regenerated silk proteins and recombinant spider silk proteins, but there are only a few reports on measured pH values in silk glands. The pH gradient in *B. mori* silk glands has been proposed to go from 6.9 in the PSG, to around 5 in the MSG and down to 4.8 in the ASG [22], but technical details for the underlying experiments were not described [17]. Another study, using injection of phenol red in the haemocoel of silkworms, showed that pH was 7–8 in the PSG and 5–6 in the MSG, while the dye could not penetrate the ASG [23]. Similar studies using pH sensitive dyes have been performed on major ampullate glands of spiders and have given pH values starting at ~7 in the sac going down to 6–6.5 in the duct [24,25]. pH sensitive dyes have low resolution, which likely explains why no difference in pH could be found between the beginning and end of the duct [24,25]. In two recent studies, pH was measured in silk glands of *B. mori* fifth instar larvae as well as in *Nephila clavipes* major ampullate glands using ion-selective microelectrodes [8,10]. The microelectrodes were very small, with tip sizes of 2–4 µm, which allowed for measurements also in the tiny PSG/tail and ASG/duct. In both the spider and the silkworm, pH was higher in the PSG/tail than previously reported, reaching >7.5 (Figure 1). In the major ampullate gland duct the pH was much lower than anticipated with a pH of 5.7 in the middle of the duct [10], while in the silkworm silk gland the lowest pH measured was 6.2 in the beginning of the ASG [8] (Figure 1). The pH gradients are apparently tightly controlled, since measured pH values are remarkably consistent, with less than 0.1 pH unit differences between individual spiders [10]. In none of the studies performed has the pH at the very end of the duct/ASG been possible to measure, since the duct/ASG is impenetrable for both dyes and microelectrodes. The exact mechanism whereby acidification of the dope occurs is still not fully elucidated. The generation and maintenance of the pH gradient has been attributed to CA activity in the ASG of silkworm silk glands [8] and in part of the sac and duct of the major ampullate gland [10] as well as to ATPase driven proton pumps in the epithelium of silkworm glands [26] and in the duct of major ampullate glands [27].

Ion concentration gradients have been suggested to play a role in fiber formation, and have been studied in both *B. mori* silk glands and major ampullate glands. In silkworms, copper levels in different parts of the silk gland were measured using proton induced X-ray emission, and an increase from PSG to ASG was measured [28]. Furthermore, proton induced X-ray emission, inductively coupled plasma mass spectroscopy and atomic adsorption spectroscopy showed that levels of sodium, potassium, magnesium and zinc also increase from PSG to ASG, while calcium levels decreased significantly [29]. Potassium, phosphorus and sulphur levels increase from proximal to distal parts of the major ampullate gland, while sodium and chloride levels decrease according to an analysis performed using cryo-SEM-EDAX (scanning electron microscope-energy dispersive X-ray) [24]. The cryo-SEM-EDAX data show statistically significant differences in ion levels at different locations, but there are large variations in the measurements, making interpretations difficult. Moreover, the final point measured is not in the duct, but in the fiber, and it is unclear if a correlation would be found if measurements were confined to the duct. For example, in *B. mori* silk glands, the copper levels were significantly higher in the fiber than in the end of the ASG [28].

The large total surface area provided by the microvilli at the apical end of the cells in the ASG/duct [8,9] probably allow for efficient absorption of water from the dope [19], which contributes to fiber formation. Shear forces are also important for fiber formation and a stress-induced phase transition of the spinning dope is evident in both spiders and silkworms [7,30,31]. Although the ASG and duct are similar in appearance and function, modeling suggests that the shear forces within the ASG and duct are different [20], with significantly stronger forces within the major ampullate gland duct, which might influence the tensile properties of the silk fibers. The difference in generated shear force between silkworms and spiders is ascribed to the differences in ASG and duct geometry [20].

## 4. A Comparison of Fibroins and Spidroins

*B. mori* fibers consist of several different proteins: the fibroin light (around 25 kDa) and heavy chain (around 350 kDa), fibroin p25 (a polypeptide which associates to the heavy and light chain by hydrophobic interactions), and sericin [32,33]. The fibroin heavy chain consists of the two hydrophilic N- and C-terminal domains (NT and CT), and a highly repetitive glycine and alanine-rich (GA) region consisting of 12 repetitive GA segments with conserved linker sequences in between. The fibroin heavy chain NT (FibNT) is 151 amino acid residues long [33] and adopts mainly a random coil conformation at neutral pH, while it folds into a double-layered anti-parallel beta sheet dimer at low pH [34]. The pH sensitivity is attributed to a cluster of acidic residues that are protonated around pH 6 and thereby the conformational change can take place [34]. The fibroin heavy chain CT (FibCT) is 58 residues long, its structure has not been characterized, and it is bound to the light chain by a disulfide bond. The fibroin light chain is a nonrepetitive protein rich in arginine and lysine. The exact function and importance of the fibroin light chain has been debated [35,36,37] but findings by Chen et al. [38] indicate that it plays an important role in lysine-mediated cross links in silk, which are common also in for example collagen and elastin.

Dragline silk also consists of several proteins, mainly major ampullate spidroins (MaSps) 1 and 2 that are very similar in primary structure [6]. The overall architecture of the MaSps is reminiscent of the fibroin heavy chain, with a repetitive region rich in glycine and alanine amino acid residues, flanked by two non-repetitive terminal domains, NT and CT. However, the amino acid sequences of NT, CT and the repetitive part are very different between the fibroin heavy chain and MaSps. The FibNT [35] and the spidroin NT (SpNT) [39] are not homologous, nor are FibCT [35] and spidroin CT (SpCT) [40]. The SpNT is an approximately 130 amino acid residue domain that folds into a five helix bundle monomer with a dipolar charge distribution at high pH, while it dimerizes and forms an antiparallel dimer at low pH [41,42,43]. In analogy to the FibNT, specific carboxylates prevent the dimerization of SpNT at high pH, and protonation of these side chains at around pH 6.5 mediates dimerization [43]. However, in contrast to FibNT, SpNT undergoes further stabilization at pH 5–6, which is mediated by protonation of a specific carboxylate [43,44]. Moreover, SpNT is alpha helical at both high and low pH, while FibNT is in random coil conformation at high pH and forms a beta sheet rich dimer structure at low pH [34,43]. The SpCT is also a five helix bundle at high pH [10,45], and is a non-covalent or covalently linked, via a disulfide bond, constitutive dimer [40]. Upon a lowering of pH, SpCT unfolds and turns into beta sheet nuclei, which may trigger further beta sheet formation of the repetitive region of spidroins (SpRep) [10,45]. Shear forces affect the SpCT in a similar way [46], and most likely pH reduction and shear forces act together to mediate SpCT conformational changes during silk formation.

There are apparent similarities between structural conversions in fibroins and spidroins since FibNT can convert to beta sheet structure at low pH [34] and SpCT unfolds and turns into amyloid-like fibrils, built up from β-sheets, upon lowering of pH [10]. However, the former transition is a bimolecular event that results in a defined soluble and globular structure, while the latter transition involves multiple intermolecular interactions and results in insoluble fibrils. The SpCT was recently found (using a web-based tool for prediction of amyloidogenic regions in proteins, waltz-switchlab.org) [47] to contain three amyloidogenic regions (Figure S1) [48], which can also be found using the Tango tool [49,50,51]. Analogous regions can be found in FibNT (Figure S1), further pointing to the possibility that FibNT and SpCT have similar functions in the control of fiber formation. Another candidate that may be responsible for nucleation dependent events in silkworm silk formation is the light chain, which also has a high amyloid-forming capacity predicted by the Waltz (Figure S1) and Tango tools.

Although the amino acid composition of the repetitive region in silkworms and spiders is quite similar, their primary structures are very different. The repetitive part of fibroins (FibRep) and SpRep are both glycine- and alanine-rich, but FibRep have (GAGAGAGS)n motifs separated by non-repetitive linker regions [35], while SpRep have alternating poly-A and glycine rich repeats, mainly GGX (as in MaSp1) or GPGXX (as in MaSp2) [52,53]. The repetitive region confers the mechanical properties to the fiber, why these differences are likely responsible for the differences seen in secondary structure and tensile properties of the fibers (cf. below). For schematic illustrations of the different spidroin and fibroin domains and their structural conversions, see for example references [1,54,55,56,57].

## 5. Are There Post-Translational Modifications of Silk Proteins?

Post-translational modifications (PTMs), such as glycosylations and phosphorylations, of silkworm silk and spider silk have been studied to some extent, but it is unclear what PTMs are present, and also the effect of these potential PTMs on fiber formation and on the mechanical properties of fibers remain to be established. MaSp1 and MaSp2 isolated from *N. clavipes* dragline silk fibers contain phosphorylation sites in their respective repetitive regions [58,59], as does FibRep [38], the fibroin light chain and the P25 [60]. The impact of these phosphorylations on the silk formation process have not been thoroughly studied, but phosphorylation in general affect soluble proteins by altering conformational states. Michal et al. [61] studied *N. clavipes* dragline silk fibers and dope using solid state 31P-NMR, and found that the dragline silk proteins contain phosphotyrosine. In another study, a phosphorylation site was included in a recombinant construct of spider dragline silk. Phosphorylation of the expressed and purified protein increased the solubility level of the construct, while dephosphorylation caused beta sheet aggregation of the protein [62]. How this relates to a possible phosphorylation of the native silk proteins has not been studied.

## 6. Protein Structure Is Different in Solution and in Fibers

We have not yet fully understood the mechanisms by which the high solubility in the spinning dopes is achieved. This feature has not been recapitulated in recombinant silk proteins or in regenerated silk, which likely is caused by the use of denaturing conditions, but additional factors may be involved as well. Both spidroins and fibroins have been hypothesized to be stored at high concentration in a soluble state in the form of protein micelles within their respective glands [21,34,55,63]. Another theory states that the dope is stored as a liquid crystalline phase [21]. These theories are not mutually exclusive and the exact state of storage of the highly aggregation prone proteins at high concentrations remains to be determined.

The structure of soluble spidroins within the lumen of the major ampullate gland is believed to be mostly alpha helical and/or random coil and some studies show that in the end of the gland, beta sheets are starting to form [25,64]. In *B. mori* the poly-GA motifs in solution (silk I) contain repeated beta-turn structures as shown by both solid-state and solution NMR [65,66], while the repeat units containing tyrosines are in random coil conformation [67]. In both silkworm and spider silk fibers, the Ala-rich regions form crystalline beta sheet structures in the fiber, while the glycine-rich regions in SpRep and the linker regions in FibRep form a more amourphous and flexible strucure based on beta spirals and random coils [68]. In silkworm silk fibers (referred to as silk II), the main structures represented are beta sheet, distorted beta turns and distorted beta sheets [69,70]. It is still unclear whether or not the beta sheets in silk fibers are parallel or antiparallel [35,71,72]. The difficulties in conclusively showing a specific structure may be related to that the silk fibers are perhaps not as highly organized as we tend to believe.

## 7. Fiber Architecture

*B. mori* fibers are 10–16 µm wide [73] while dragline silk fibers are significantly thinner, around 3–6 µm [74]. The *B. mori* fiber consists of two fibroin monofilaments originating from the two separate glands, with a coat of sericin [73,75]. The architecture of dragline silk fibers has been debated, and the fiber has been proposed to consist of two to five layers. A skin-core structure has been suggested [9,76,77,78], but also four-layered [79] or five-layered structures [80] have been proposed. The lack of conclusive data about the architecture of dragline silk probably relate to the fact that these studies involve extensive treatment of the silk, such as dehydrating, embedding in plastic, sectioning and staining [9,77,78], dipping silk fibers in urea and allowing them to supercontract [79] or treating fibers using ether, triton x-100 and freeze-thaw cycles, after which they were embedded and sectioned [80]. Most studies have, however, agreed upon the presence of micro- and nanofibrils in parallel to the fiber axis within the dragline silk [78,80,81,82,83], which is also the case for silkworm silk [75,83,84].

The presence of nano- and micro-fibrils within the fiber could be important for its tensile properties. Twisted microfibrils would significantly increase the toughness of the fiber, a feature which is frequently utilized in manmade fibers and ropes. Relaxation studies on native spider silk has pointed towards a torsional memory in dragline silk [85,86], which may in part explain the high toughness of spider silks. One aim of the production of artificial fibers has been to get fibers that are as homogenous as possible, an ambition that may be questionable since native dragline silk fibers are apparently quite heterogenous. A certain heterogeneity of the structure within the fiber may increase the strength of the fiber, while too much or too little heterogeneity could cause fibers to be less strong [87].

## 8. Tensile Properties of Silk Fibers—Why Is Spider Silk Tougher?

**Figure 3:**
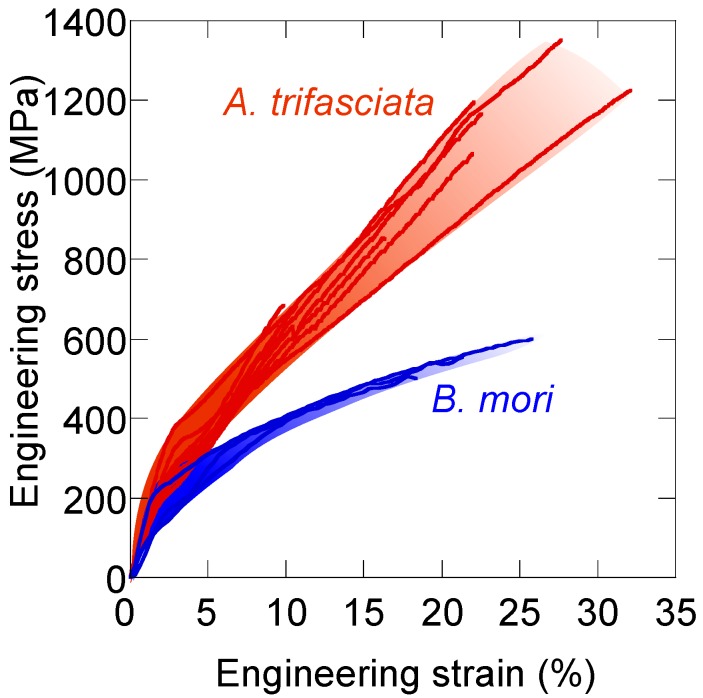
Stress vs. strain curves of forcibly silked native dragline silk fibers from *A. trifasciata* (in red) and silk fibers from *B. mori* (in blue). Original data from [88,89].

Fibers spun by spiders and silkworms both have a very high toughness, compared to other natural and manmade fibers, but spider silk is in general superior to silkworm silk. The stress/strain behaviors show that the dragline silk from *Argiope trifasciata* is much stronger (higher maximum stress before breaking) and also more extendible (higher strain) than *B. mori* fibers (Figure 3).

**Figure 4:**
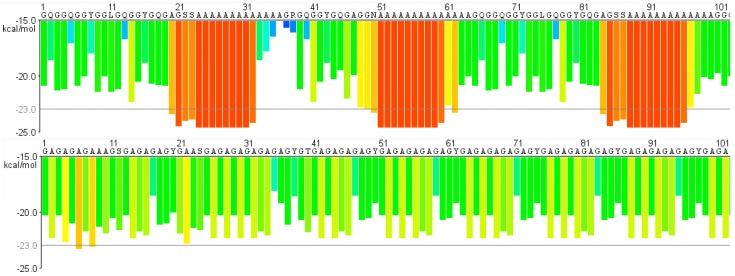
Fibrillation propensity profiles of the first 100 amino acid residues of *B. mori* fibroin heavy chain repetitive part (AF226688.1) (**top**) and three repetitive blocks from *E. australis* MaSp1 (GenBank AM490183) (**bottom**). The amino acid residues are presented in one-letter format above each plot. The energies are color-coded, blue and green representing higher energies while orange and red represents lower energies that are deemed to have high fibrillation propensity. Profiles generated by ZipperDB [93].

There are likely several factors that lead to spider silk being tougher than silkworm silk, and several models of how the toughness is mediated have been put forward, e.g., [57,68,90,91,92]. One hypothesis states that the presence of poly-GA repeats in the repetitive part of fibroins, as compared to poly-A blocks present in spidroins, will create a lower binding strength of the beta sheets [68], which likely also affects the tensile properties. Hayashi and co-authors modelled that the poly-GA will form less stable beta sheets than poly-A since Gly lacks a side-chain that can mediate hydrophobic interactions [68]. Comparing the binding strength of potential beta sheets of SpRep and FibRep using the Zipper database [93], which can be used to predict amyloid fibril forming segments, one can see that the energies of the poly-GA motifs in the FibRep are much higher than those of the poly-A blocks in SpRep (Figure 4), which means that the poly-GA motifs are predicted to form much weaker beta sheets. This observation further argues that the nature of the repetitive parts to a large extent explains the lower toughness exhibited by silkworm silk.

Neither the poly-GA nor the poly-A segments of the repetitive regions are intrinsically prone to forming beta sheets. In fact, Ala is the naturally occurring residue with the highest α-helical propensity and Gly is disfavoured in any secondary structure [94]. This may be a prerequisite for keeping the proteins in solution before spinning, and any other stretch of amino acid residues could pose a danger by being prone to aggregate prematurely [95].

## 9. Conclusions and Future Perspectives

By comparing silk spinning in two distantly related species, in which the ability to spin silk apparently evolved after they separated during evolution [5,6], we find some features that appear to be crucial for successful fiber formation. Firstly, the pH gradient is tightly controlled, is present in both silk production systems, and mediates dramatic secondary structure transitions of the silk proteins, via specific effects on the terminal domains; Secondly, the silk proteins are highly soluble at neutral pH. The high overall solubility may be attributed to the highly soluble terminal domains, but also the repetitive regions rich in alanine and glycine residues are water soluble, in contrast to stretches of hydrophobic residues [96]; Thirdly, silk proteins are large, >300 kDa, which may be necessary in order to obtain the great mechanical strength and extensibility displayed by the fibers, although alternative explanations to the large sizes, based on general genetic mechanisms, are also possible [97].

We believe that using a biomimetic approach, wherein silk proteins are not denatured throughout the purification and spinning processes, is vital for the generation of artificial silk fiber replicas with the same structure and toughness as native silk fibers. Supporting this are results from experiments on silkworm silk extracted from cocoons that are solubilized using high temperatures and lithium bromide [98] before spinning fibers, which results in silk that is very different in structure and inferior in toughness compared to native silkworm silk [88,99,100] likely due to the denaturation of the native silk proteins. In contrast to silkworms, spiders produce very small amounts of silk, and therefore largescale production of spider silk fibers requires expression in and purification from heterologous hosts. Expression of highly repetitive sequences such as the MaSp repetitive region is difficult in heterologous hosts, and therefore most constructs have been considerably smaller than the native spidroins, often including only a small part of the repetitive region and lacking one or both of the terminal domains. The water solubility levels of such constructs have still been very low (around 1% *w*/*v* [101,102]), and recombinant spidroins have therefore been dissolved in denaturing agents (reaching solubility levels of 5%–25% *w*/*v* [103,104,105]), after which spinning into tough fibers has proven difficult. In the same way that regenerated (denatured) silkworm silk cannot form fibers that are similar to native silk, it is highly unlikely that artificial fibers spun from denatured recombinant spidroins can capture the true structure and toughness of native spider silk.

Although the physiology and biochemistry of silk spinning has been studied quite thoroughly over the past decade, there is apparently a lot that we still do not understand. For example, spidroins and fibroins produced in the epithelial cells are stored in secretory granula. How are these extremely pH sensitive and aggregation-prone proteins able to withstand the presumably quite low pH within the secretory granules without starting to assemble? What chaperone systems are involved in successfully transporting the silk proteins through the secretory pathway, a process that is demanding even for “normal” proteins [106]? We look forward to further detailed studies of silk proteins and the silk formation process, which can generate insights that have broad and important implications.

## Supplementary Materials

Supplementary materials can be found at www.mdpi.com/1422-0067/17/8/1290/s1.

## Abbreviations

PSG	Posterior silk gland
MSG	Middle silk gland
ASG	Anterior silk gland
CA	Carbonic anhydrase
Cryo-SEM-EDAX	Cryo scanning electron microscope energy dispersive X-ray
NT	N-terminal domain
CT	C-terminal domain
FibNT	Heavy chain fibroin N-terminal domain
FibCT	Heavy chain fibroin C-terminal domain
MaSp	Major ampullate spidroin
SpNT	Spidroin N-terminal domain
SpCT	Spidroin C-terminal domain
SpRep	Spidroin repetitive region
FibRep	Heavy chain fibroin repetitive region
PTM	Post-translational modification
NMR	Nuclear magnetic resonance
