# Supplementary material for: Silk Spinning in Silkworms and Spiders
Source: Int J Mol Sci. 2016 Aug 9;17(8):1290. doi: 10.3390/ijms17081290 (PMC5000687; doi:10.3390/ijms17081290)
Supplement: Supplementary file 1 [file ijms-17-01290-s001.pdf]

Supplementary Materials: Silk Spinning in Silkworms and Spiders

Marlene Andersson, Jan Johansson and Anna Rising

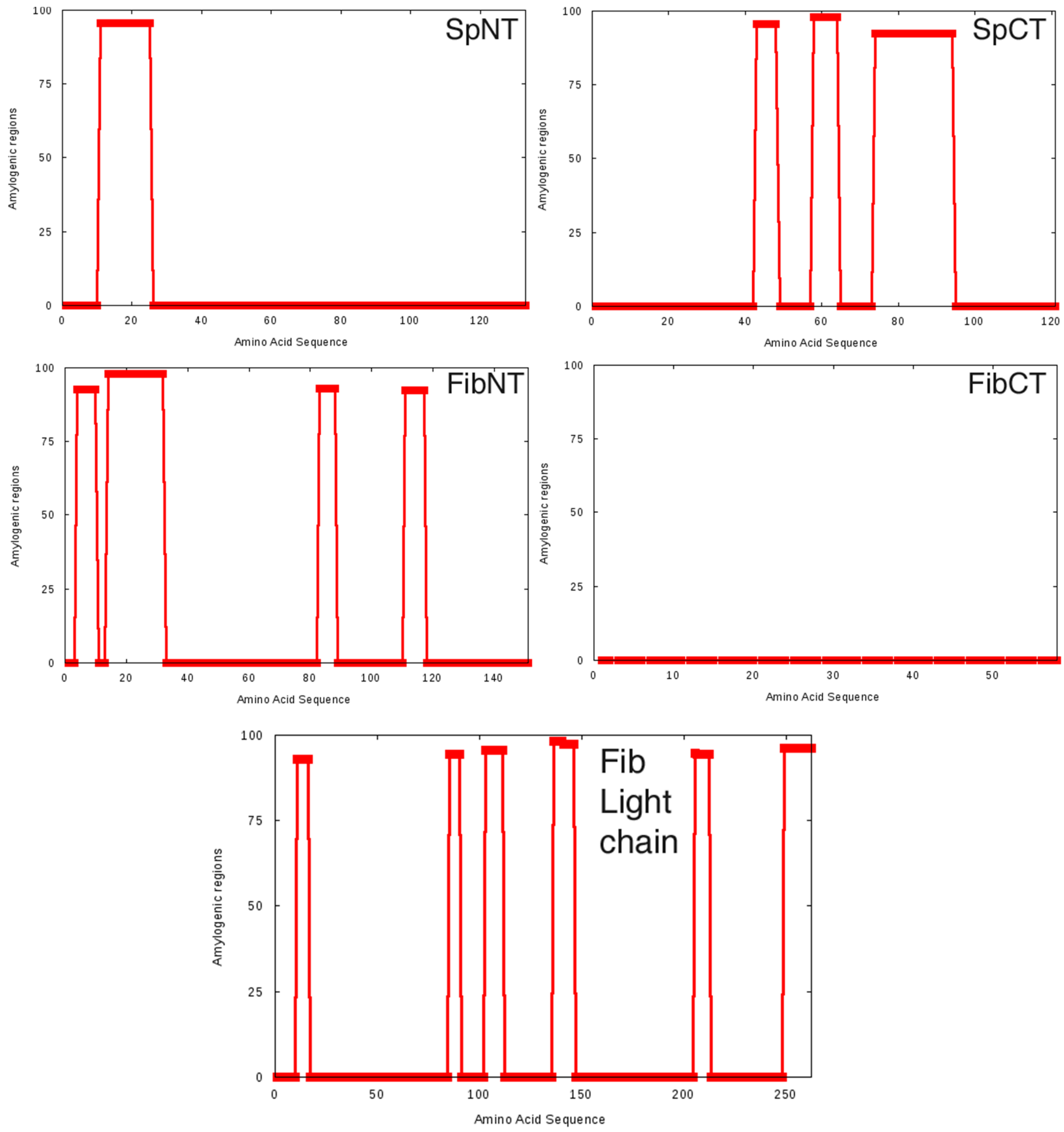

Figure S1. Waltz plots of spidroin NT (SpNT), spidroin CT (SpCT), fibroin heavy chain NT (FibNT), fibroin heavy chain CT (FibCT) and fibroin light chain.
